# The Effect of Additives on the Early Stages of Growth of Calcite Single Crystals

**DOI:** 10.1002/anie.201706800

**Published:** 2017-08-18

**Authors:** Yi‐Yeoun Kim, Colin L. Freeman, Xiuqing Gong, Mark A. Levenstein, Yunwei Wang, Alexander Kulak, Clara Anduix‐Canto, Phillip A. Lee, Shunbo Li, Li Chen, Hugo K. Christenson, Fiona C. Meldrum

**Affiliations:** ^1^ School of Chemistry University of Leeds Woodhouse Lane Leeds LS2 9JT UK; ^2^ Department of Materials Science and Engineering University of Sheffield Sheffield S1 3JD UK; ^3^ Current address: Materials Genome Institute Shanghai University Shanghai 200444 China; ^4^ School of Mechanical Engineering University of Leeds Woodhouse Lane Leeds LS2 9JT UK; ^5^ Institute of Microwaves & Photonics School of Electronic & Electrical Engineering University of Leeds Leeds LS2 9JT UK; ^6^ School of Physics and Astronomy University of Leeds Leeds LS2 9JT UK

**Keywords:** biomineralization, calcite, crystal growth, microfluidics

## Abstract

As crystallization processes are often rapid, it can be difficult to monitor their growth mechanisms. In this study, we made use of the fact that crystallization proceeds more slowly in small volumes than in bulk solution to investigate the effects of the soluble additives Mg^2+^ and poly(styrene sulfonate) (PSS) on the early stages of growth of calcite crystals. Using a “Crystal Hotel” microfluidic device to provide well‐defined, nanoliter volumes, we observed that calcite crystals form via an amorphous precursor phase. Surprisingly, the first calcite crystals formed are perfect rhombohedra, and the soluble additives have no influence on the morphology until the crystals reach sizes of 0.1–0.5 μm for Mg^2+^ and 1–2 μm for PSS. The crystals then continue to grow to develop morphologies characteristic of these additives. These results can be rationalized by considering additive binding to kink sites, which is consistent with crystal growth by a classical mechanism.

Soluble additives are widely used to generate crystals with defined morphologies, sizes, and structures. However, owing to the difficulty of studying dynamic phenomena on the nanoscale, surprisingly little is known about the effects of additives on the initial growth of crystals. Monitoring the development of crystals by isolating them from solution at different reaction times is challenging as crystals initially change rapidly in size and can undergo dissolution and regrowth. The best insight into the growth of small crystallites has probably come from the study of metal^1^ and metal oxide nanoparticles,^2^ which terminate at small particle sizes and have well‐defined morphologies. In situ analytical methods are particularly promising as demonstrated in a recent study of the surfactant‐directed growth of Pd nanocubes by liquid‐phase transmission electron microscopy (LP‐TEM).^3^

We herein made use of a fascinating phenomenon, namely that crystallization processes are retarded in small volumes,^4^ to investigate the effects of the contrasting soluble additives Mg^2+^ and poly(styrene sulfonate) (PSS) on the early stages of growth of calcite single crystals formed from an amorphous calcium carbonate (ACC) precursor phase. With its rich polymorphism, CaCO_3_ provides a much‐studied model system that is also biologically, environmentally, and industrially important. Techniques such as time‐resolved wide/small‐angle X‐ray scattering^5^ and light scattering^6^ have been used to study the precipitation pathway of CaCO_3_ in bulk solution while cryo‐TEM has provided snapshots of its development.^7^ LP‐TEM has also been used to follow the precipitation of CaCO_3_ in additive‐free solutions,^8^ and in the presence of PSS.^9^ However, little is known about the initial morphological development of calcite crystals in the presence of additives. This study addresses this challenge by using a “Crystal Hotel” microfluidic device^10^ as a simple and versatile means of investigating the morphological development of crystals. Each device provides an array of independent “rooms” with volumes of just 19 nL (Figure 1) that not only slow down the rate of crystal growth, but also avoid the problems associated with impurities, incomplete mixing, and convection that are often found in bulk solution. Some adsorption of PSS on poly(dimethylsiloxane) (PDMS) can be expected, but this has a negligible effect at the additive concentrations used. Studying CaCO_3_ precipitation within the Crystal Hotel provides strong evidence that the crystallization of ACC in solution begins at the surface of the ACC particles, and that ACC can be transformed directly into calcite, in contrast to previous suggestions.^8^ Surprisingly, the morphological changes characteristic of Mg^2+^ and PSS are not observed until the crystals reach sizes of at least 100 nm, which we rationalize with a simple model based on classical crystal growth and additive binding to kink sites.

**Figure 1:**
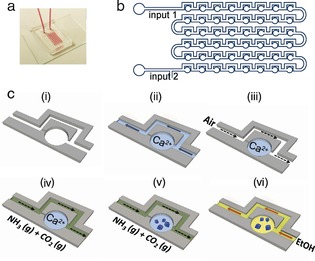
The Crystal Hotel microfluidic device. a) Photograph of a PDMS device bonded to a glass slide and filled with a solution of red dye. b) Chip design of a Crystal Hotel with 48 “rooms”. c) Crystallization in a single room (i). An aqueous solution (light blue) is introduced through inlet 2 to fill the channel and rooms (ii). Subsequently, air (white) is introduced through inlet 1 to push the solution out of the channel and isolate the solution contained in each room (iii). (NH_4_)_2_CO_3_ vapor (green) is then pumped through inlet 1 (iv), and CaCO_3_ precipitation is initiated by diffusion of CO_2_ and NH_3_ gas into the solution (v). Once crystals have formed, ethanol (yellow) is pumped through the device to terminate the reaction (vi).

Crystal Hotel devices were created from PDMS using common lithographic methods and bonded to a glass slide. Based on a design by Sun and co‐workers,^11^ each device has a sequence of 48 cylindrical rooms with a diameter of 400 μm and a height of 150 μm, which are linked by microchannels (Figure 1). The rooms were filled with CaCl_2_ solution ([Ca^2+^]=1.25–5 mm) containing either [Mg^2+^]=0–5 mm or [PSS]=0–500 μg mL^−1^ (*M*_w_=70 000), and then CaCO_3_ precipitation was induced by introducing (NH_4_)_2_CO_3_ vapor at 50 μL min^−1^.^12^ The estimated range of supersaturations is given in the Supporting Information, Figure S1. The reactions were allowed to proceed for 5–30 min, and were then terminated by circulating ethanol through the device (Figure 1 c). The particles deposited on the glass slide in the Crystal Hotel were then characterized by scanning electron microscopy (SEM) and Raman microscopy (Figure S2) after removal of the PDMS layer.

ACC was the first phase formed and subsequently transformed into calcite at different times depending on the nature of any additives present. The transformation into calcite occurred within 5 min in the absence of additives, as compared with 20 min at [Ca^2+^]=1.25–5 mm and [Mg^2+^]/[Ca^2+^]≥1 and about 10 min at [Ca^2+^]=1.25–5 mm and [PSS]=250–500 μg mL^−1^ (Figures 2 a–d and Figures S3 and S4). Both PSS and Mg^2+^ ions are known to retard the transformation of ACC into crystalline polymorphs,^13^, ^14^ which has been attributed to an inhibition of the nucleation and growth of the new crystalline phase rather than a direct effect on the ACC.^13^ As crystallization processes are retarded within small volumes, it was possible to use the Crystal Hotel to observe the transformation mechanism. A large proportion of ACC hemispheres formed in the presence of [Mg^2+^]=1.25 mm at [Ca^2+^]=2.5 mm exhibited small rhombohedral particles, morphologically consistent with calcite, on their surfaces (Figures 2 a, b). This provides strong evidence that the transformation of ACC into calcite is initiated adjacent to the ACC/solution interface, and that the calcite crystallites then grow at the expense of the amorphous material. ACC particles imaged by SEM prior to the onset of crystallization showed no evidence of surface calcite crystallites, showing that they do not result from drying/irradiation (Figure S5).

**Figure 2:**
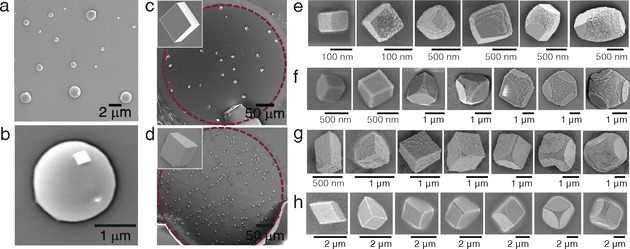
SEM images of CaCO_3_ precipitates in the Crystal Hotel. a, b) Amorphous calcium carbonate obtained at [Ca^2+^]=2.5 mm and [Mg^2+^]=1.25 mm. Further images of ACC precipitated under a range of solution conditions are shown in Figures S3 and S4. c, d) Calcite crystals grown without additives at [Ca^2+^]=5 mm and [Ca^2+^]=50 mm, respectively. The purple dotted line indicates the boundaries of the individual “room”. The insets show crystals at higher magnifications. e–h) Crystals formed the presence of e) [Ca^2+^]=2.5 mm and [Mg^2+^]=1.25 mm, f) [Ca^2+^]=2.5 mm and [PSS]=500 μg mL^−1^, g) [Ca^2+^]=2.5 mm and [PSS]=250 μg mL^−1^, and h) [Ca^2+^]=1.25 mm and [PSS]=250 μg mL^−1^.

The transformation mechanism of ACC into calcite has been the subject of much discussion, and dissolution/recrystallization, a direct solid‐state transformation, or even a combination of mechanisms^15^ have been suggested. ACC can also be directly transformed into calcite or via a vaterite intermediary phase.^5^ Recent LP‐TEM studies support the existence of multiple nucleation pathways,^8^ and aragonite and vaterite crystals have been seen to form in direct contact with ACC particles, with nucleation of the crystalline phase occurring adjacent to the surface of an ACC particle. However, no evidence for a direct transformation of ACC into calcite had been obtained, which led to the suggestion that this mechanism is unlikely.^8^ A recent study of the transformation of ACC in picoliter droplets on SAMs, in contrast, indicated a direct transformation of ACC into calcite.4d The hypothesis that ACC crystallization is initiated at the ACC/water interface was also supported by another recent study, which showed that ACC dehydrates before crystallizing, even in solution, and that the loss of the final water fraction coincides with crystallization.15a The high activation energy of the final step is in keeping with partial dissolution/recrystallization. The fact that we here observed rhombohedral crystallites on the surfaces of the ACC provides intriguing support for the latter mechanism, while this is also consistent with simple arguments based on the relative magnitudes of the interfacial free energies. Contact between a hydrated calcite surface and water should be more favorable than that between a calcite surface and ACC, where little water of hydration is available.

We then focused on the morphological development of the nascent calcite crystals and were surprised to observe that none of the morphological signatures associated with Mg^2+^ or PSS were observed until the crystals were much larger in size (Figure 2 e–h). The mature crystals did, however, display morphologies comparable to those of their counterparts precipitated in bulk solution (Figure S6). In the case of Mg^2+^, the mature crystals exhibited roughened surfaces and an elongation along the *c* axis, which is due to the interaction of the Mg^2+^ ions with acute step edges.^16^ At [Ca^2+^]=2.5 mm and [Mg^2+^]=1.25 mm, the typical “transition size” at which morphological changes were first observed was 0.1–0.5 μm (Figure 2 e). Analogous results were obtained for the polymeric additive PSS. At [Ca^2+^]=2.5 mm and [PSS]=500 μg mL^−1^, deviations in morphology involving truncations of the corners and edges only became apparent when the crystals attained sizes of approximately 1 μm. This effect became more pronounced as the crystals grew (Figures 2 f) until morphologies comparable to those obtained in bulk solution were obtained (Figure S6); these comprise rhombohedra with roughened surfaces and truncated edges that are flattened perpendicularly to the *c* axis.^17^ In contrast to Mg^2+^, PSS preferentially interacts with the obtuse steps on calcite, causing the difference in morphology. Ca^2+^ concentrations of ≥5 mm or higher additive concentrations resulted in polycrystalline particles (Figures S6 and S7).

The impact of the reaction conditions on the crystal sizes at which morphological changes were observed was also explored. A reduction in the concentration of PSS from 500 μg mL^−1^ to 250 μg mL^−1^ while holding [Ca^2+^] constant at 2.5 mm had little effect on the transition size or the final crystal morphology (Figure 2 f vs. g), where this trend was also observed in bulk solution (Figure S6). In contrast, an increase in the transition size from about 1 μm to approximately 2 μm was observed upon reducing [Ca^2+^] from 2.5 mm to 1.25 mm at [PSS]=250 μg mL^−1^ (Figure 2 g vs. h). These results are thus consistent with our assertion that the additives had little effect on the morphologies of the calcite crystals at sizes below 100 nm.

In interpreting our experimental data, it is important to note that as in the majority of studies of additive/crystal interactions, the Crystal Hotel offers a “batch” system in which the solution composition varies as ions are consumed. We therefore performed experiments to confirm that this was not the origin of the effects seen in our study. During crystal growth, the concentration of Ca^2+^ decreases significantly, while there is little change in the additive concentrations (as they are only sparingly incorporated into the calcite crystals). The additive/Ca^2+^ ratio therefore increases during crystal growth and reaches very high levels shortly before growth terminates.^18^ Rough calculations showed how the solution composition changes during crystallization (Figure 3 a). Assuming a final population of 3 μm crystals, only 1.6 % of the Ca^2+^ ions had been consumed when the crystals reached a transition size of 0.5 μm, and for a transition size of 1 μm, this value is still only 3.7 %. There is thus little change in the solution composition at the point of the first changes in morphology. We also performed experiments under continuous‐flow conditions to confirm that the initial additive concentrations were sufficient to induce changes in morphology. Exposure of 1–3 μm calcite seed crystals to a supersaturated solution with constant composition yielded product morphologies consistent with those observed for micrometer‐sized crystals in the Crystal Hotel (Figure 3 b and c). Finally, we monitored the growth of crystals within the Crystal Hotel rooms by optical microscopy (Movie S1). This showed that the crystals form over a short time period rather than over a prolonged period of time as is often observed in the bulk. This highlights one of the key advantages of the Crystal Hotel over bulk solutions: it offers well‐defined reaction environments in which a uniformly supersaturated solution rapidly forms.

**Figure 3:**
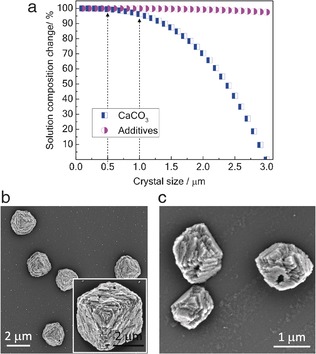
a) Estimated change in the solution composition during crystallization from a solution with [Ca^2+^]=2.5 mm and [PSS]=250 μg mL^−1^. b, c) Crystals grown from 1–3 μm calcite seed crystals in a continuous‐flow cell from a solution with a constant composition of [Ca^2+^]=3.2 mm, [HCO_3_^−^]=6.4 mm, [NaCl]=35 mm, and [PSS]=250 μg mL^−1^. Ionic strength=0.05 mm, pH 8.5, SI=1.4.

Our understanding of how additives affect the morphology of crystals such as calcite has mainly come from macroscopic specimens.^19^ In situ atomic force microscopy (AFM) studies have revealed how calcite growth at the nanoscale is influenced by soluble additives, and have shown how Mg^2+^ ions interact with the step edges on calcite.^16^, ^20^ Macroscopic calcite crystals are generally rhombohedral, bounded by low‐energy {104} faces. Growth occurs via screw dislocations at supersaturations lower than about twice the saturation, and by surface nucleation and subsequent 2D growth at higher concentrations.^21^ Both of these structures exhibit acute and obtuse step edges to which additives may selectively bind,^16^, ^20^ and it is recognized that the growth of low‐solubility crystals such as calcite is limited by the availability of kink sites.^22^ Strong additive binding to the kink sites leads to changes in the shape, separation, and velocity of the step edges, which can ultimately cause a pile‐up of steps, resulting in rugged faces, which define the crystal habit.^19^

Our experiments show the dominance of the {104} faces, even at small crystal sizes. The crystal size at which dislocations form in calcite is not known, however.^23^ The efficacy of an additive in controlling crystal growth depends on its residence time at a kink site compared to the step propagation rate. For a very small crystal, which necessarily exhibits short step lengths and hence few kink sites, the probability of an impurity binding and affecting the completion of that growth layer is very small. As the crystal increases in size, the area of the faces, the length of the step edges, and the number of kink sites increase (Figures 4 a–c). This increases the chance that an additive will retard the propagation of step edges and the completion of growth layers. Smaller transition sizes are therefore expected for more strongly binding additives (Figure 2 e vs. f), as observed. Higher supersaturations give rise to a greater density of step edges,^20^, ^22^ and thus smaller transition sizes (Figure 2 f vs. h).

**Figure 4:**
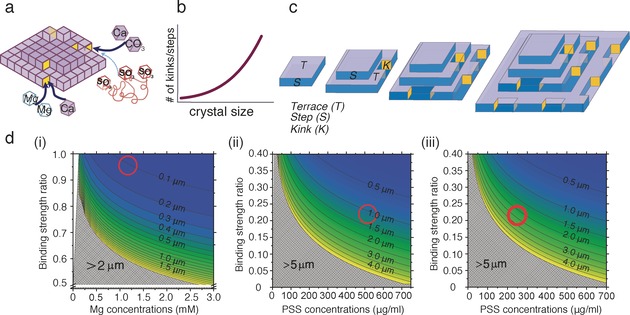
Schematic summary of how additives affect calcite morphology. a) Mg^2+^ and PSS compete with the Ca^2+^ and CO_3_^2−^ growth units to bind to kink sites (yellow) at step edges. The thickness of the arrows indicates the relative binding strengths. b) The length of the step edges, and hence the number of kink sites, increases with the crystal size. c) For simplicity, only one {104} face is shown. Newly formed calcite crystals have few kink sites, and the probability of additive binding is low. As the crystals grow, an increase in the length of the step edges and the number of associated kink sites raises the probability of additive binding. Ultimately, the crystals are sufficiently large for additive binding to cause a change in the macroscopic crystal shape. d) “Transition sizes” at which morphological changes are predicted as a function of the additive/Ca^2+^ binding strength ratios at kink sites. The red circles show our experimental data for i) [Ca^2+^]=2.5 mm and [Mg^2+^]=1.25 mm, ii) [Ca^2+^]=2.5 mm and [PSS]=500 μg mL^−1^, and iii) [Ca^2+^]=1.25 mm and [PSS]=250 μg mL^−1^.

Support for this model was obtained from simple calculations estimating the fraction of kink sites on a calcite crystal that are occupied by additive molecules rather than CaCO_3_ units, as a function of the crystal size. This is calculated from the product of the molarity ratio of additive to CaCO_3_ unit and the binding energy ratio of additive to CaCO_3_ unit. Therefore, binding to a kink site increases with additive concentration and/or binding energy. Finally, we assume that a morphological change will occur when at least one kink site is occupied by an additive. These calculations were carried out for Mg^2+^ and PSS based on the known frequency of steps and kink sites on the calcite {104} surface (see the Supporting Information). Figure 4 d shows that as the concentration of additives in solution and their binding strength increases, the transition size associated with a morphology change decreases. Mg^2+^ has a similar binding strength to Ca^2+^,^24^ and both ions will have similar concentrations at the kink sites. Mg^2+^ ions are therefore expected to affect the morphology as soon as sufficient kink sites are present. At a [Mg^2+^]/[Ca^2+^] ratio of 1:2 (Figure 4 d (i), red circle), the model predicts a morphological change at crystal sizes of about 100 nm, in excellent agreement with our experimental data.^25^ The fact that Mg^2+^ ions affect crystal morphology at smaller particle sizes than PSS is also consistent with the effects of these additives on bulk crystal growth, where Mg^2+^ ions are strongly inhibitory owing to the combined effects of kink blocking and the enhanced solubility of Mg‐substituted calcite.^16^, ^22^

Given the lack of data on the binding of PSS to calcite, it is more difficult to make predictions for PSS. However, rough estimates can be made based on simulations of the binding of methanoic acid and poly(acrylic acid) (PAA) to calcite, where the free energy of binding for Ca^2+^ and methanoic acid to a {104} face was estimated to be −7 kJ mol^−1^ 25a and −1.58 kJ mol^−1^,25b respectively. The fact that polymeric additives adsorb and modify crystal habits much more strongly than the corresponding monomers was considered using simulations that showed that about 15 % of the functional groups of PAA were bound to calcite at any given time.^26^ Hence, 58 of the 385 sulfonate groups in each of the PSS chains used here would be expected to bind. Concentrations of 250 μg mL^−1^ and 500 μg mL^−1^ PSS are approximately equivalent to 0.18 mm and 0.36 mm of styrene sulfonate (SS) side groups, respectively. Additionally, as the kink sites are far apart, each polymer chain can only bind to a single kink site. We have indicated this region with a red circle in Figures 4 d (ii) and (iii), which predicts morphological changes at crystal sizes of 1 μm at [PSS]=500 μg mL^−1^ and [Ca^2+^]=2.5 mm, and 2 μm at [PSS]=250 μg mL^−1^ and [Ca^2+^]=1.25 mm. These values are again close to our experimental ones.

These results demonstrate that confinement provides an effective strategy for slowing down, and thus studying, crystallization processes. By using a Crystal Hotel microfluidic device, we have obtained strong evidence for the direct transformation of ACC into calcite in solution and have shown that calcite crystals growing in the presence of Mg^2+^ and PSS are perfect rhombohedra until their size reaches at least 100 nm and 1 μm, respectively. The size at which an additive begins to affect the morphology of calcite depends on the additive binding strength, the concentration, and the supersaturation, which was rationalized by considering additive binding to available kink sites. Our data also confirm that calcite grows by a classical ion‐by‐ion mechanism in the presence of PSS^18^ rather than a non‐classical assembly of nanoparticles.^27^ These results provide insight into the growth mechanisms of sparingly soluble crystals such as calcite, and show that it is important to consider the action of additives on nucleation and growth to obtain product crystals with the desired properties.

## Conflict of interest

The authors declare no conflict of interest.

## Supporting information

As a service to our authors and readers, this journal provides supporting information supplied by the authors. Such materials are peer reviewed and may be re‐organized for online delivery, but are not copy‐edited or typeset. Technical support issues arising from supporting information (other than missing files) should be addressed to the authors.

Supplementary

Supplementary
