# Supplementary material for: The Effect of Additives on the Early Stages of Growth of Calcite Single Crystals
Source: Angew Chem Int Ed Engl. 2017 Aug 18;56(39):11885–90. doi: 10.1002/anie.201706800 (PMC5638089; doi:10.1002/anie.201706800)
Supplement: Supplementary file 1 — Supplementary [file ANIE-56-11885-s001.pdf]

Angewandte Chemie
Eine Zeitschrift der Gesellschaft Deutscher Chemiker

Supporting Information

The Effect of Additives on the Early Stages of Growth of Calcite Single Crystals

Yi-Yeoun Kim, Colin L. Freeman, Xiuqing Gong, Mark A. Levenstein, Yunwei Wang, Alexander Kulak, Clara Anduix-Canto, Phillip A. Lee, Shunbo Li, Li Chen, Hugo K. Christenson, and Fiona C. Meldrum**

anie_201706800_sm_miscellaneous_information.pdf
anie_201706800_sm_Movie_SI1_travelodge.avi

1. Experimental Methods

1.1 Materials.

Analytical grade $(NH_4)_2CO_3$, $CaCl_2 \cdot 2H_2O$, $MgCl_2 \cdot 6H_2O$, poly-styrene sulfonate (PSS Mw 70,000) and Styrene-4-sulfonic acid sodium salt (SS) were purchased from Sigma-Aldrich and were used as received. Polydimethylsiloxane (PDMS) structures were prepared using a SYLGARD 184 silicon elastomer kit from Dow corning. All solutions were prepared using Milli-Q deionized water (18.2 MΩ cm^{-1}). Glass slides were used after cleaning by placing in Piranha solution (70:30 vol% H_2SO_4: H_2O_2) overnight and then washed copiously with Milli-Q water followed by ethanol, before drying with air.

1.2 Microfluidic Chip Fabrication

1.2.1 Fabrication Silicon/Resist Template. Polydimethylsiloxane (PDMS) was selected as the bulk material as it is an inexpensive silicon rubber which is flexible, transparent and biocompatible. The fabrication method of our chip is described as follows. Firstly, a silicon wafer was cleaned at 70 ^{o}C in a solution of (NH_4OH : H_2O_2 : H_2O = 1 : 1 : 5) for 15 min, followed by DI water washing, compressed N_2 gas drying and baking in an oven at 120 ^{o}C for 30 min. Photoresist SU-8 2050 was then spin-coated onto the wafer at 1000 rpm. After soft baking, the wafer was exposed to UV light through a photomask, hard baked and finally developed in SU-8 developer to obtain the desired mold pattern for the flow channels as shown in Fig.1.

1.2.2 Fabrication of Crystal Hotel. PDMS pre-polymer was prepared by mixing silicone elastomer base and curing agent at a weight ratio of 10 : 1. The mixture was then poured onto the silicon wafer and solidified in a 60 ^{o}C oven for 2h. The solidified PDMS containing the embedded fluid channel structures was removed from the substrate, and holes for outlets were punhced, as shown in Fig. 1b. After treatment with 0.5M NaOH for 10 mins to hydrolyze the surface, the PDMS slab was sealed to another glass slide by plasma bonding.

1.3 $CaCO_3$ Precipitation and Analysis

1.3.1 $CaCO_3$ Precipitation in Bulk. Calcium carbonate was precipitated using the ammonium carbonate diffusion method[15], where $CaCO_3$ was precipitated in the presence of Mg^{2+} ions and PSS polymers in 12 MicroWell™ Plates containing total 2 mL of aqueous solution. Glass substrates were cleaned by soaking in Piranha solution prior to use as described above, and were placed at the base of the each well. Stock solutions of $[Ca^{2+}]$ = 20 mM, $[Mg^{2+}]$= 20 mM and [PSS] = 10 mg/ml) and [SS] = 200 mM were mixed with a solution of $[Ca^{2+}]$ = 1.25 – 5 mM to form final solutions with Mg^{2+} concentrations of 0-5 mM, SS

concentrations of 0–100 mM and PSS concentrations of 0 – 500 μg mL^{-1}. Precipitation of $CaCO_3$ was initiated by placing the dish in a sealed desiccator containing a glass Petri dish of $(NH_4)_2CO_3$ (3-5 g), which was covered with a Parafilm with holes. Crystallization was typically allowed to proceed for 1 day (unless stated otherwise). Following this period, the glass slides supporting the $CaCO_3$ crystals were removed from the solutions, were washed with Milli-Q DI water and/or ethanol, and were dried in an oven at 40 ^{o}C.

1.3.2 $CaCO_3$ Precipitation in Crystal Hotel Chips. The chip was pre-run for 3-5 mins with an aqueous solution of the desired concentration of calcium chloride and additives using a 5 mL syringe mounted on a syringe pump (Harvard Apparatus, flow rate: 50 μL min^{-1}) to fill all of the channels and rooms with solution and remove any trapped air. Pre-filling with water prior to using the reaction solutions was avoided as it can cause a Ca^{2+} concentration gradient to form in the rooms. Another 5 mL syringe filled with air was plunged quickly (flow rate 100-200 μL min^{-1}) to remove solution from the flow channel (Figs 1b-1c) while trapping and isolating the solution in each "hotel" room. Subsequently, a third 5 mL syringe filled with 1 g of ammonium carbonate powder was pumped into open air for 5 min to allow time for the powder to decompose. The syringe was then connected to the chip and the NH_4 and CO_2 vapors were slowly pumped into the chip at 20 μL min^{-1} for 1 – 30 min. To quench the reaction and rinse the room, another 5 mL syringe filled with ethanol was pumped for 5 mins from inlet 2. The PDMS was then carefully removed using a Stanley 28-510 Razor Blade and the substrate with exposed rooms was kept in a petri-dish and dried with air. Analysis of the hotel rooms showed that solution trapped in the first row of rooms often evaporated relatively quickly, so were excluded from further examination. Rooms in the last row were also excluded, since the diffusion of gas into these rooms occurs much more slowly, such that neither crystals nor amorphous material could be observed in this row under the reaction times studied here.

1.3.3 $CaCO_3$ Growth in a Continuous Flow Cell. 1-3 μm calcite seed crystals were formed from a 2 mM Ca^{2+} solution on pre-cleaned microscope slides using the ADM method as described above. The glass slide supporting the seed crystals was then used in the construction of a flow cell, where a Teflon insert with aperature (10mm in diameter) was sandwiched between two glasses (one with seed crystals, and the another clean slide). The device has one inlet and one outlet, and silicon tubing is used to flow solution into, and out of the cell. The pre-made supersaturated solution contained [$CaCl_2$] = 3.2mM, [NaCl] = 35 mM, [$NaHCO_3$] = 6.4 mM, pH = 8.5 and [PSS] = 250 μg ml^{-1} and was used to fill the cell, and then was flow continuously at 300 μL min^{-1} using a syringe pump for 3 hours. Fresh solution was employed every 30 mins.

1.3.4 Characterization of $CaCO_3$ Particles. Precipitated $CaCO_3$ particles were analysed using Optical microscopy, Scanning Electron Microscopy (SEM) and Raman microscopy. The crystals on glass slides were examined uncoated using a CBS detector using an FEI Nova NanoSEM™. Raman microscopy was employed to further confirm the polymorph of individual particles grown in bulk, where this was carried out using a Renishaw 2000 Raman microscope operating with a 785 nm diode laser.

1.4 Model of Additive Binding to Kink Sites

The growth modelling was carried out using a custom-developed code. The code assumes that a kink site must be occupied by either a Ca^{2+}/CO_3^{2-} ion or the additive, and calculates the fraction of sites occupied by each. This result is then multiplied by the number of kink sites on the particle and if the number of kink sites occupied is 1 or greater, morphological changes are assumed to occur. The fraction of sites occupied by the additive, θ, is calculated as,

$$\theta = f \ \times population(\theta)$$

where *f* is the collision frequency (i.e. the chance of the additive reaching the kink) and *population (*θ*)* is the number of kink sites that would be occupied by the additive at the thermodynamic minimum. We assume that only Ca^{2+}/CO_3^{2-} ions or the additive can bind at the kink site and both species have the same dynamics in the solution. Therefore, the collision frequency, *f*, is simply the relative molar ratio of the two species; the more additive that is present, the more likely it is to reach the kink site than the Ca^{2+}/CO_3^{2-} ions. The population is calculated via statistical mechanics assuming the two states possible are the kink occupied by Ca^{2+}/CO_3^{2-} ions (E_{ions}) or the additive (E_{add})

$$population(\theta) = \frac{\mathrm{e}^{\frac{-E_{add}}{k_B T}}}{\mathrm{e}^{\frac{-E_{add}}{k_B T}} + \mathrm{e}^{\frac{-E_{ions}}{k_B T}}}$$

where k_B is the Boltzmann constant and T is the temperature (298 K). The values of E_{add} are defined as a fraction of E_{ions}. A kink site was assumed to occur every 25 nm on surface steps for 2.5 mM of Ca^{2+} and 50 nm for 1.25 mM of Ca^{2+}, based on published experimental data examining step density as a function of supersaturation [32].

In the case of PSS adsorption, the code was modified to account for the high degree of binding of the PSS molecule at the surface (but not necessarily at the kink site) which would lead to a higher local concentration of PSS at the surface than the basic molarity would suggest. The

value of *f* was calculated as a combination of the likelihood of PSS binding in the surface region versus Ca^{2+}/CO_3^{2-} ions, where this was calculated using

$$f = \frac{58\, M_{add}}{M_{ions}} \times \frac{\mathrm{e}^{\frac{-E_{add}}{k_B T}}}{\mathrm{e}^{\frac{-E_{add}}{k_B T}} + \mathrm{e}^{\frac{-E_{ions}}{k_B T}}}$$

where M_{add} is the molarity of the PSS, M_{ions} is the molarity of the Ca^{2+}/CO_3^{2-} ions and E_{add} = 2.75 E_{ions} (due to the greater binding strength of the whole PSS molecule compared to the individual ions). The additional factor of 58 was added to represent the number of functional groups available for binding to the kink after the PSS has bound to the particle surface. The value of 58 was chosen as molecular weight estimates of the PSS used here suggest that ≈ 385 functional groups would be present in the average PSS molecule and previous simulations of large molecules have suggested binding of long chain molecules at a calcite surface may involve 15% of the total functional groups [29]. Note that the *population* (θ) part of the equation used the same values for E_{adds} and E_{ions} as used for the SO_3 ions.

Figure SI1. Estimated supersaturation levels of the reaction solutions employed, where these were determined using Visual Minteq. Supersaturation index(SI) is defined as $\log\sigma = a_{Ca^{2+}}\, a_{CO_3{}^{2-}}/K_{sp}$. σ_{acc} , $\sigma_{vaterite}$ and $\sigma_{calcite}$ indicate supersaturation with the respect to amorphous calcium carbonate ($K_{sp,\ acc}$ = $10^{-6.393}$), vaterite ($K_{sp,vaterite}$ = $10^{-7.93}$) and calcite ($K_{sp,calcite}$ = $10^{-8.48}$). Data is not available to explicitly calculate the contribution of PSS to the solution supersaturation, such that estimates were made based on the equivalent concentration of sulfonate functional groups.

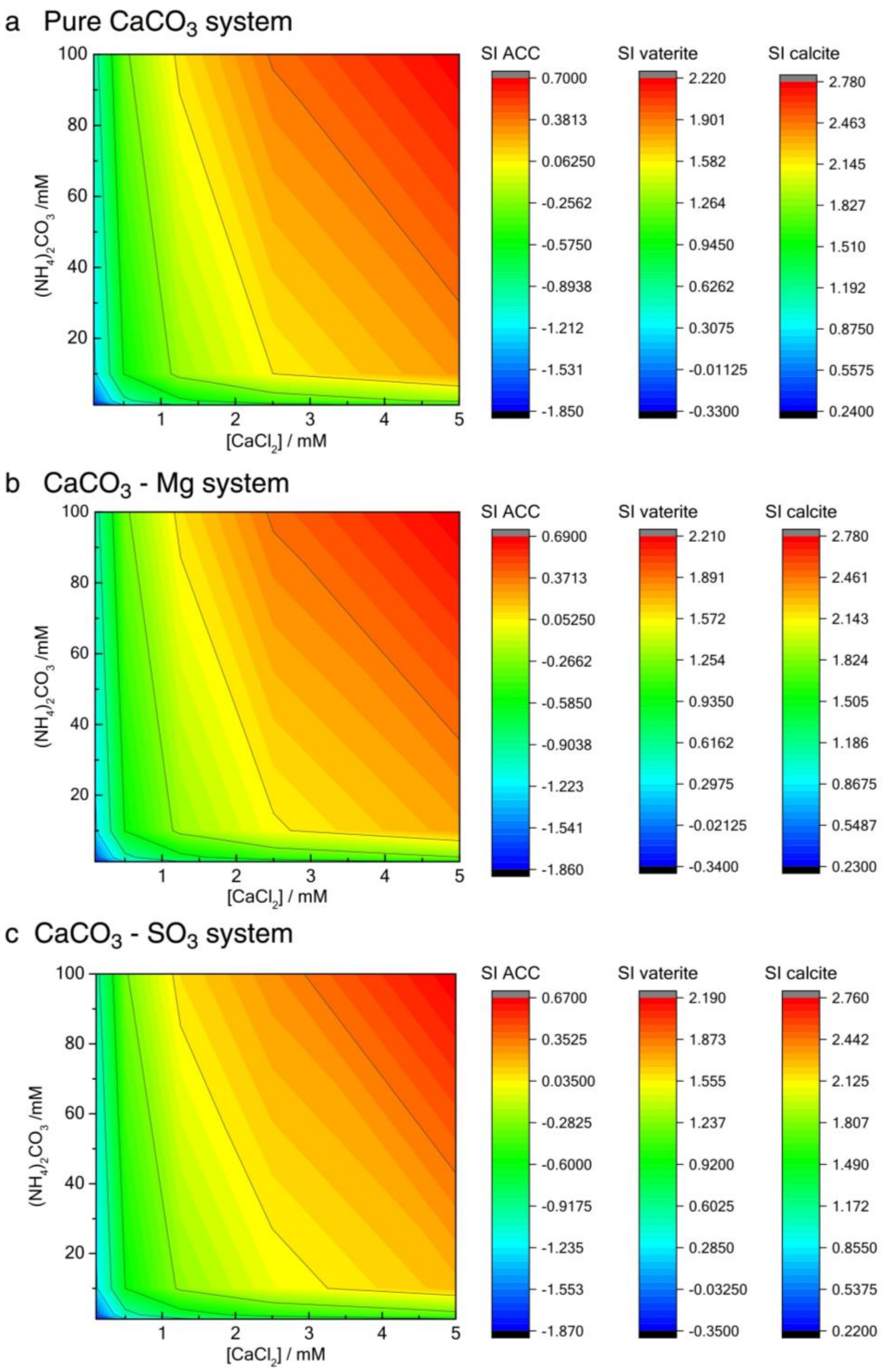

Figure SI2. Raman spectra of crystals precipitated in bulk solutions in the presence of (a) Mg^{2+} and (b) PSS. All spectra show characteristic peaks of calcite, where the v_1 (1085 cm^{-1}) and v_4 (711 cm^{-1}) bands can be assigned to the CO_3^{2-} symmetric stretch and symmetric bend respectively, and two lattice mode peaks labeled with red arrows are also shown at 159 and 281 cm^{-1}.

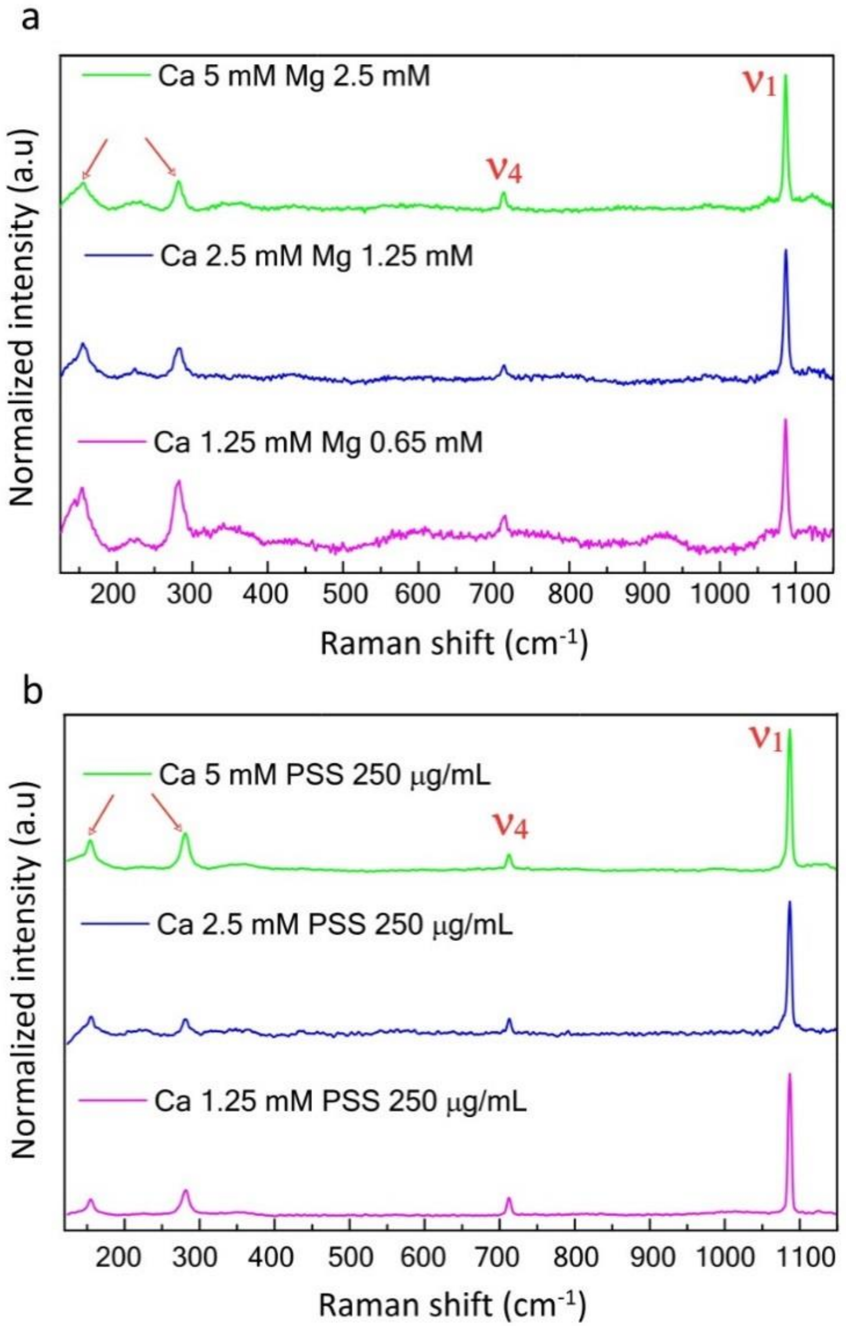

Figure SI3. SEM images of ACC or its transition to crystalline obtained in the Crystal Hotel at the early stages of precipitation in the presence of additive (a-b) at $[Ca^{2+}]$ = 1.25 mM and $[Mg^{2+}]$ = 1.25 mM (c-d) at $[Ca^{2+}]$ = 2.5 mM and $[Mg^{2+}]$ = 1.25 mM (e) at $[Ca^{2+}]$ = 5mM and $[Mg^{2+}]$ = 2.5 mM and (f) at $[Ca^{2+}]$ = 5mM and $[Mg^{2+}]$ = 5 mM.

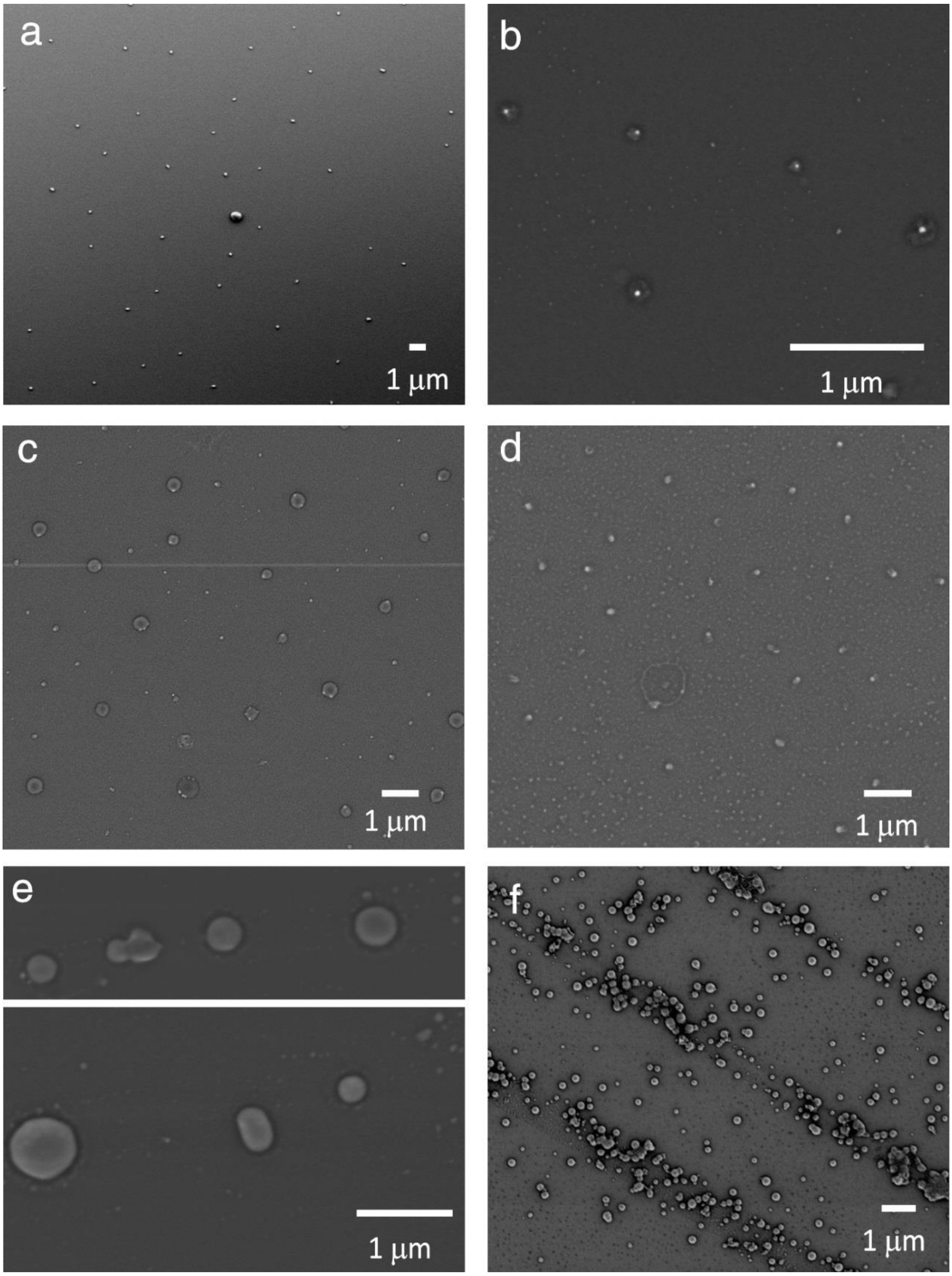

Figure SI4. SEM images of amorphous calcium carbonate (ACC) or transition to crystalline obtained in the Crystal Hotel at the early stages of precipitation in the presence of additive PSS (g and h) at $[Ca^{2+}]$ = 1.25 mM and [PSS] = 250 µg mL^{-1}. (i and j) at $[Ca^{2+}]$ = 1.25 mM and [PSS] = 500 µg mL^{-1}. (k and l) $[Ca^{2+}]$ = 2.5 mM and [PSS] = 250 µg mL^{-1}. (m and n) $[Ca^{2+}]$ = 5 mM and [PSS] = 500 µg mL^{-1}.

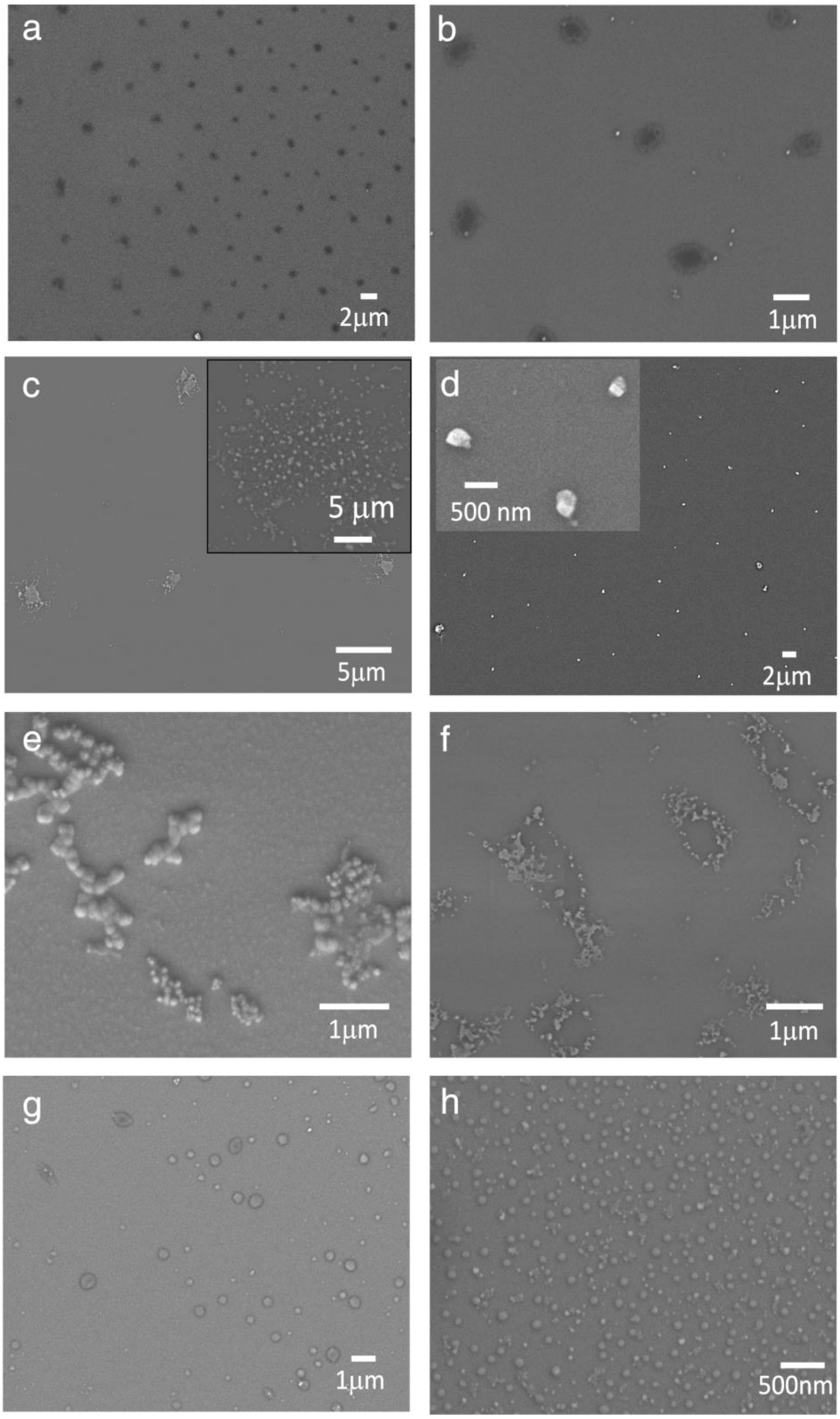

Figure SI5. SEM images of ACC precipitated in the Crystal Hotel under conditions [Ca^{2+}] = 2.5 mM and [Mg^{2+}] = 1.25 mM (where these are identical to the condition used to produce the samples shown in Figures 2c-d (b) [Ca^{2+}] = 5 mM and [Mg^{2+}] = 2.5 mM. Dehydration of ACC in the SEM during long irradiation with the electron beam generates pores. This confirms that the nucleation of calcite on the surfaces of the ACC particles was not caused by drying or electron beam irradiation.

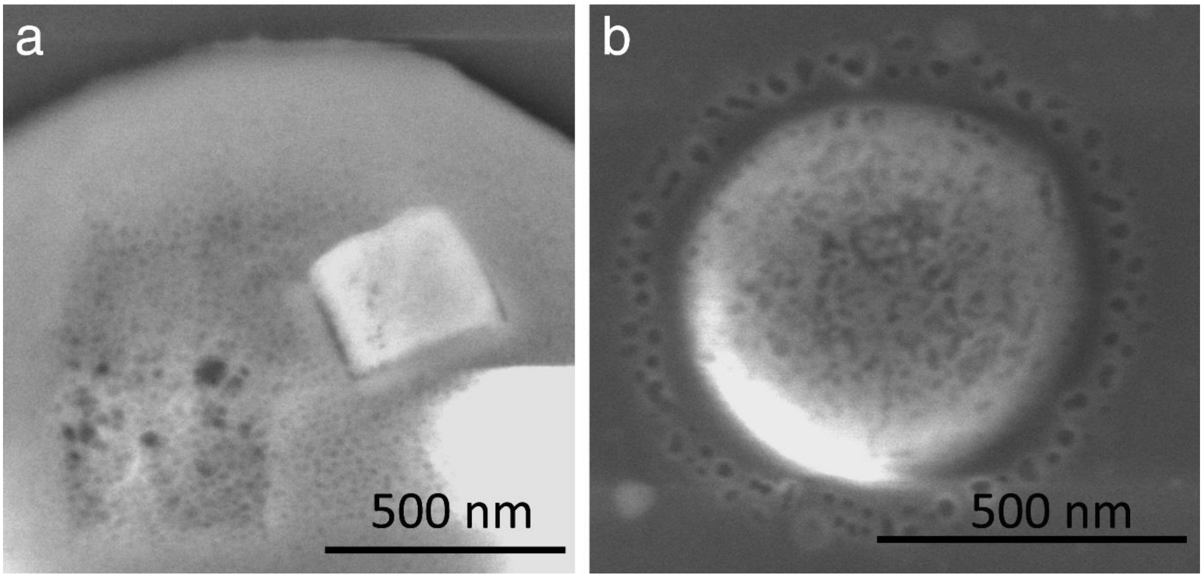

Figure SI6. (a) SEM images of calcite crystals precipitated in bulk solution at a range of Ca^{2+} (rows) and Mg^{2+} concentrations (columns). Increasing the [Mg^{2+}] has a greater effect on calcite morphology than increasing [Ca^{2+}]. (b) SEM images of calcite crystals grown in bulk solution at a range of Ca^{2+} (rows) and PSS concentrations (columns). In this system, increasing [Ca^{2+}] has a greater morphological effect than increasing [PSS].

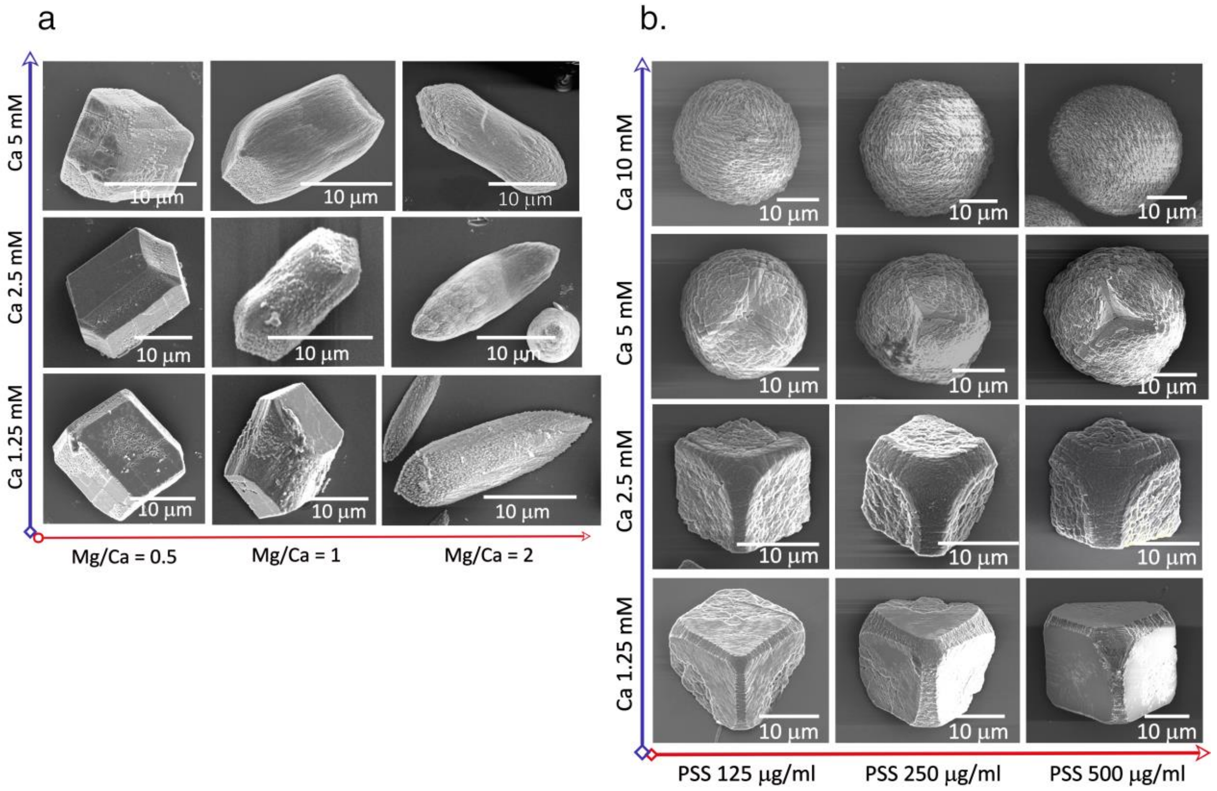

Figure SI7. (a) SEM images of calcite crystals precipitated in the Crystal Hotel under conditions [Ca^{2+}] = 5 mM and [Mg^{2+}] = 2.5 mM. (b) SEM images of precipitates in the Crystal Hotel under conditions [Ca^{2+}] = 5 mM and [PSS] = 250 μg mL^{-1}. Polycrystalline calcite, comparable to the crystals obtained in bulk solution (Figure S11c), appeared after 20 min.

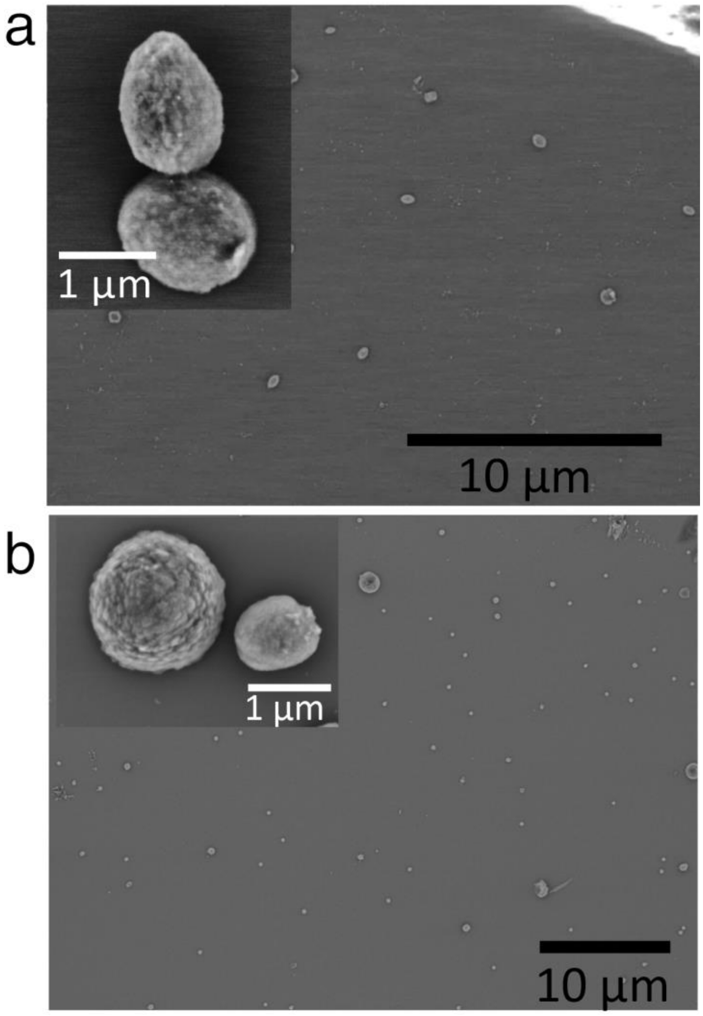

Movie SI1. Movie of $CaCO_3$ crystallization in a room in the Crystal Hotel under solution conditions [Ca^{2+}] = 2.5 mM and [PSS] = 250 μg mL^{-1} . It showed that most crystals nucleated at the same time and then grew until the Ca^{2+} ions in the solution were depleted.
